# Endometrial regenerative cells for treatment of heart failure: a new stem cell enters the clinic

**DOI:** 10.1186/1479-5876-11-56

**Published:** 2013-03-05

**Authors:** Leo Bockeria, Vladimir Bogin, Olga Bockeria, Tatyana Le, Bagrat Alekyan, Erik J Woods, Amalia A Brown, Thomas E Ichim, Amit N Patel

**Affiliations:** 1 Bacoulev Institute for Cardiovascular Surgery, Moscow, Russia; 2 Medistem Inc, San Diego, California, USA; 3 Cook General Biotechnology, Indianapolis, USA; 4 Department of Medicine, University of Utah, Salt Lake City, Utah, USA

## Abstract

Heart failure is one of the key causes of morbidity and mortality world-wide. The recent findings that regeneration is possible in the heart have made stem cell therapeutics the Holy Grail of modern cardiovascular medicine. The success of cardiac regenerative therapies hinges on the combination of an effective allogeneic “off the shelf” cell product with a practical delivery system. In 2007 Medistem discovered the Endometrial Regenerative Cell (ERC), a new mesenchymal-like stem cell. Medistem and subsequently independent groups have demonstrated that ERC are superior to bone marrow mesenchymal stem cells (MSC), the most widely used stem cell source in development. ERC possess robust expansion capability (one donor can generate 20,000 patients doses), key growth factor production and high levels of angiogenic activity. ERC have been published in the peer reviewed literature to be significantly more effect at treating animal models of heart failure (*Hida et al. Stem Cells 2008*).

Current methods of delivering stem cells into the heart suffer several limitations in addition to poor delivery efficiency. Surgical methods are highly invasive, and the classical catheter based techniques are limited by need for sophisticated cardiac mapping systems and risk of myocardial perforation. Medistem together with Dr. Amit Patel Director of Clinical Regenerative Medicine at University of Utah have developed a novel minimally invasive delivery method that has been demonstrated safe and effective for delivery of stem cells (*Tuma et al. J Transl Med 2012*). Medistem is evaluating the combination of ERC, together with our retrograde delivery procedure in a 60 heart failure patient, double blind, placebo controlled phase II trial. To date 17 patients have been dosed and preliminary analysis by the Data Safety Monitoring Board has allowed for trial continuation.

The combined use of a novel “off the shelf” cell together with a minimally invasive 30 minute delivery method provides a potentially paradigm-shifting approach to cardiac regenerative therapy.

## Introduction

Cell-based approaches to heart failure are grounded in the concept that regeneration is mediated by the administered cells themselves and/or that the cells act as producers of trophic factors which stimulate cardiac reparative processes such as angiogenesis and expansion of endogenous cardiac specific stem cells [1,2].

In 2001, clinical use of cell therapy in cardiac disease was reported by three independent groups using autologous myoblast [3] and bone marrow mononuclear cells [4,5]. The promising results of these pilot studies led to formal trials, which demonstrated statistically significant, albeit small, improvements in cardiac function. For example, a meta-analysis of 4 randomized controlled studies [6-9] and 2 cohort studies [10,11] evaluating autologous bone marrow cells administered transepicardially during coronary artery bypass graft (CABG) revealed a 5.4% increase in left ventricular ejection fraction (LVEF) in a total of 179 patients [12]. Bone marrow cells administered via the intracoronary route were analyzed in 8 clinical trials in post-infarct patients [13-22]. A meta-analysis of the cumulative 725 patients revealed increased LVEF by 4.37% and reduction in left ventricular end-diastolic volume (LVEDV) by 5.71 mL, left ventricular end-systolic volume (LVESV) by 8.94 mL, and infarct size by 2.42%, which were all statistically significant [23].

Bone marrow mesenchymal stem cells (MSC) have also been developed as a cell source for regenerative cardiology. One advantage of MSC based approaches is that they may be used as “universal donor” cells that function across different HLA haplotypes. This allogeneic approach is favored from a commercialization perspective in that cells from a single donor may be used to produce banks of cells that may be frozen and distributed to the point of care. Bone marrow MSC have demonstrated therapeutic effects in post infarct patients subsequent to intravenous administration, thus increasing practicality of the approach. In a 53 patient trial, the global symptom score in all MSC treated patients and ejection fraction was significantly improved as compared to placebo. Additionally MSC treatment, but not placebo, increased left ventricular ejection fraction and led to reverse remodeling [24]. Another study compared bone marrow mononuclear cells with allogeneic bone marrow MSC in 30 patients with ischemic cardiomyopathy. At one year endpoint adverse events were similar between the two groups, with a mild increase in ejection fraction [25].

The possibility of using MSC-based therapies for heart failure is enticing. MSC have the ability to directly differentiate into cardiomyocytes [26,27], as well as to secrete angiogeneic and trophic factors [28-30], which assist in regeneration and possibly activation of endogenous cardiac stem cells [31]. MSC appear to be immune privileged and immune modulatory. Specifically, they are poor stimulators of allogeneic immunity and in many cases have been shown to actively inhibit ongoing immune responses [32,33].

Besides the practical applications of MSC being capable of transport and storage at the site of care in the same manner that a drug would be, MSC may be standardized and optimized for specific cytokine/regenerative activities. This is useful in that autologous cells from patients with underlying conditions appear to function sub-optimally as compared to age-matched control cells. For example, it has been demonstrated that angiogenic potency of bone marrow from patients with coronary artery disease is impaired, in part due to deficiencies in the CXCR4 migration activity [34].

### Endometrial regenerative cells: a novel type of MSC

The endometrium is a unique tissue in that it undergoes approximately 500 cycles of highly vascularized growth and regression within a tightly controlled manner in the lifetime of the average female. This has triggered the concept that a self-renewing cell capable of differentiating into various tissues may be present in the endometrium. The possibility of a regenerative cell type resident in the endometrium was proposed by Prianishnikov in 1978, who reported that three types of proliferative cells exist: estradiol-sensitive cells, estradiol- and progesterone-sensitive cells and progesterone-sensitive cells [35]. Interestingly, a study in 1982 demonstrated that cells in the endometrium destined to generate the decidual portion of the placenta are bone marrow derived [36], which prompted the speculation of a stem cell-like cell in the endometrium. Further hinting at the possibility of stem cells in the endometrium were studies demonstrating expression of the immortality associated gene telomerase in endometrial tissue collected during the proliferative phase [37,38]. One study demonstrated that telomerase expression was upregulated by estradiol and FGF-2, however this was restricted to epithelial cells of the endometrium [39]. Expression of stem cell markers such as c-kit and the pluripotency marker Oct-4 was reported in proliferating “label retaining” cells of the endometrium, thus further supporting the concept that stem cells exist in this compartment [40].

One of the first reports of proliferative cells from the endometrium identified clonogenic cells capable of generating stromal and epithelial cell colonies, however no differentiation into other tissues was reported [41,42]. The phenotype of these cells was found to be CD90 positive and CD146 positive [43]. The cells isolated by this group appear to be related to maintaining structural aspects of the endometrium but to date have not demonstrated therapeutic potential.

The first demonstration of therapeutically-relevant stem cells derived from the endometrium occurred almost simultaneously by two independent groups. The Medistem group [44], used the process of cloning rapidly proliferating adherent cells derived from menstrual blood and generated a homogenous cell population expressing CD9, CD29, CD41a, CD44, CD59, CD73, CD90, and CD105 and lacking CD14, CD34, CD45 and STRO-1 expression. The authors demonstrated the cells had substantially faster replicative potential as compared to bone marrow MSC, a unique cytokine and MMP profile, as well as ability to differentiate into cardiomyocytic, respiratory epithelial, neurocytic, myocytic, endothelial, pancreatic, hepatic, adipocytic, and osteogenic lineages. Interestingly, the cells identified expressed telomerase and OCT-4 but lacked expression of NANOG-1. Given the pluripotent nature of these cells, the authors named them “Endometrial Regenerative Cells” (ERC). Shortly after, Patel et al. [45] reported a population of cells isolated using c-kit selection of menstrual blood mononuclear cells. The cells had a similar phenotype, proliferative capacity, and ability to be expanded for over 68 doublings without accumulation of karyotypic abnormalities. Interestingly, both groups found expression of the pluripotency gene OCT-4 but not NANOG.

### Therapeutic efficacy of ERC

Given the high degree of angiogenesis occurring monthly in the endometrium, it is tempting to speculate that ERC possess a physiological role in this process. This is supported by the high concentrations of angiogenic factors and MMPs expressed in ERC compared to other MSC types [44]. The therapeutic angiogenesis promoting cytokine, VEGF, is naturally found in the endometrium and its production is stimulated monthly by estradiol [46]. Further supporting a role for ovary-derived hormones in endometrial vasculogenesis were studies in which ovarectomy resulted in lack of VEGF production [47] and angiogenesis [48]. While the cellular mechanisms of angiogenesis appear to be multifactorial and involve neutrophils [49,50], uterine NK cells [51], and circulating endothelial progenitor cells [52-54], the possibility of ERC playing a significant role in this process may be considered.

To assess the role that ERC play in angiogenesis, Murphy et al. create an aggressive murine hindlimb ischemia model comprising of femoral artery ligation with nerve excision. ERC administration was capable of reducing limb loss in all treated animals, whereas control animals suffered limb necrosis [55]. Given that the ERC were of human origin and the treated animals were immune competent (BALB/c), it is tempting to speculate that the cells possess a high level of immune privilege. This was supported by experiments demonstrating inhibition of ongoing mixed lymphocyte reaction, stimulation of the anti-inflammatory cytokine IL-4 and suppression of inflammatory cytokine (IFN-g and TNF-alpha) production.

The relationship between angiogenesis and post myocardial infarct healing is well-known. Given previous work by Umezawa’s group demonstrating myocytic differentiation of ERC-like cells [56], administration of ERC into a model of post infarct cardiac injury was performed [57]. Recovery was compared to bone marrow MSC. A superior rate of post-infarct recovery of ejection fraction, as well as reduction in fibrosis was observed with the ERC-like cells. Furthermore, the cells were capable of functionally integrating with existing cardiomyocytes and exerted effects through direct differentiation. The investigators also demonstrated in vitro generation of cardiomyocytes from ERC.

The stimulation of neurogenesis in post-stroke injury models has been demonstrated to be linked directly to angiogenesis [58]. Borlongan et al. [59], injected ERC intracerebrally, or intravenously into immune competent rats subsequent to middle cerebral artery ligation. They demonstrated substantial increases in functional recovery of the rats receiving ERC. Additionally, it was shown that ERC secreted significant amounts of neurotrophic growth factors including VEGF, BDNF, and NT-3.

A recent study from David Stroncek’s group at the NIH compared gene expression profiles between bone marrow MSC and ERC generated from 6 donors [60]. Angiogenic cytokines PDGF-BB and angiopoietin were expressed at 27-fold and 14-fold higher levels in the ERC compared to bone marrow MSC, respectively. Furthermore, matrix metalloprotease-3 was expressed at 29-fold higher levels in ERC. These data provide confirmation of previously published results in which protein levels of these factors were found to be higher in ERC compared to bone marrow MSC and cord blood MSC [44]. Of note, in the NIH study, significantly higher expression of the stem cell potency-associated gene aldehyde dehydrogenase (39 fold), as well as immune modulatory genes such as pregnancy associated glycoprotein 1 (30 fold), neuronal pentatraxin (15 fold), GM-CSF (5 fold) and DAF (3 fold) was observed. The heightened expression of immune modulatory genes by ERC as compared to bone marrow MSC is supported by enhanced ability to inhibit mixed lymphocyte reaction [60].

### Preclinical and clinical safety of ERC

From a safety perspective, one original concern was the development of ectopic endometriosis. Recent studies have demonstrated that the cells capable of producing endometrosis-like masses in NOD-SCID mice are of the fibroblast lineage and do not appear to be related to ERC. To address this potential concern Medistem performed acute (14 day) and chronic (90 day) toxicity/tumorigenicity studies of human ERC in male and female immunocompromised mice. Cells were prepared according to the protocol for clinical supply. Groups of mice (both sexes) received a single unilateral intramuscular (IM) injection into the gastrocnemius muscle. Agents tested included a control article (saline vehicle), a positive control fibrosarcoma cell line and a low, medium and high dose of ERC (3 × 10^4^, 1 × 10^5^, and 2 × 10^5^, respectively).

Observations were made throughout the study. Body weights were measured at intervals in the study. Organ weights and blood chemistry/hematology were measured at the time of necropsy. Also at necropsy, gross pathologies were noted and tissues were collected for histopathological analysis. No early deaths occurred in the ERC-treated or control groups. All animals in the positive control groups receiving IM injections of the fibrosarcoma cell line HT1080 developed tumors by 3 weeks and either died (1 mouse) or were sacrificed because of excessive tumor pathology. No toxicological trends or biologically significant differences, including body weight, were observed and values were in the normal range of historic data. Histopathological examination of the injection site revealed no abnormalities. Therefore, based on these data, a single intramuscular injection of ERC at doses bracketing the intended clinical dose appear to be safe and well tolerated in immunocompromised mice.

Due to the highly angiogenic nature of ERC, one potential concern would be the possibility of stimulating progression of dormant tumors. Accordingly, Medistem performed a series of experiments in the C6 model of rat glioma to assess whether intervenous or intratumoral injection of ERC would modulate tumor growth. Substantial inhibition of growth was observed by both intravenous (49% reduction compared to control) and intratumoral (46% reduction compared to control) groups [61]. These data suggest that ERC actually act as inhibitory cells for tumor growth.

The first report of clinical use of ERC involved 4 patients with multiple sclerosis who received both intrathecal and intravenous injections [62]. Patients received a series of 3–5 injections with a total dose of 16–30 million cells. No physical, biochemical, or radiological abnormalities were observed at follow up. The patients, who were treated in 2008, have reported no adverse events at time of last follow-up (September 2012). The second published report described a 23 year old male diagnosed with Duchenne Muscular Dystrophy. On August 5–14 and November 25–28, 2008 the patient was treated a total dose of 116 million ERC intramuscularly. No reactions were associated with the stem cell infusion. As of August 1^st^ 2011, no treatment associated adverse events were noted and the patient was in general good health [63]. The third clinical report described a 74-year old heart failure patient who received a total of 15 million ERC intravenously over the period of a week. No adverse events were reported. The last follow-up was April 1^th^, 2010 and the patient being in good health [64].

### A New delivery technique for stem cells in heart failure: retrograde administration into the coronary sinus

Historically cardiac cell delivery techniques included intramyocardial administration (transepicardially or transendocardially) or antegrade delivery into the coronary artery. The transepicardial route suffers the drawback of surgical invasiveness. The transendocardial approach requires complex electromagnetic mapping using systems such as the NOGA device. Additionally, intramyocardial administration is not applicable for patients with thinned myocardium, as this technique may cause perforation. Antegrade administration into the coronary artery is associated with a lower number of cells engrafting [65], as compared to intramyocardial administration. This is due to the fact that once the administration balloon is deflated under the high arterial pressure; the administered cells dislodge and enter the systemic circulation. Additionally, administration of cells into the coronary artery has been shown to increase risk of coronary embolism and ST segment elevations [66-70].

In humans the coronary sinus drains the anterior left ventricular wall, which is responsible for the majority of cardiac contraction. The technique of retrograde delivery into the coronary sinus has been widely used for the administration of cardioplegia solution due in part to superior distribution of the solution throughout the myocardium as compared to antegrade delivery [71]. Additionally, the procedure has been demonstrated clinically safe for administration of oxygenated blood during high risk percutaneous transluminal coronary angioplasty [72-74].

The process of retrograde administration into the coronary sinus involves temporary occlusion of afferent coronary circulation by means of a balloon catheter followed by administration against the outflowing blood. This results in the solution entering the myocardium via post capillary venules. In contrast to arterioles or capillaries, post-capillary venules have the smallest vessel diameter and conceptually would allow for greatest transfer of material into the interstitium [75]. Physiologically, it is known that post-capillary venules are a major target of immune/inflammatory cell migration across the endothelium, in part due to expression of adhesion molecules such as ELAM-1 [76], ICAM-1 [77], CD18 [78], and CD44 [79], and in part because of biomechanical properties. Given that MSC [80,81], hematopoietic stem cell [82], and various tissue specific progenitors [83,84], migrate into tissue using similar mechanisms/molecules of extravasation as activated leukocytes, it is reasonable to directly deliver cells to “exit ports” within the coronary microcirculation as compared to intra-arterially.

Several studies not involving cell therapies have successfully utilized retrograde administration in the area of cardiac regeneration. Boekstegers et al. [85], delivered adenovirus expressing beta-gal and Luciferase into the porcine myocardium comparing antegrade delivery into the coronary artery or retrograde via the anterior cardiac vein. Significantly elevated expression of the gene in infarct tissue in a homogenous manner was observed via the retrograde method as compared to antegrade. Similar results were reported by Alino et al. [86], who observed interstitial expression of eGFP in porcine hearts that were injected in the retrograde manner with naked DNA. In another study, administration of beta-gal encoding plasmid using the retrograde method in pigs resulted in higher myocardial gene expression in comparison to antegrade and intramuscular administration [87]. This superior level of gene expression in comparison to intramyocardial delivery was reproduced in other studies [88].

The use of retrograde administration has also been performed successfully for delivery of protein therapeutics. von Degenfeld et al. [89], reported a porcine study in which retrograde administration of FGF-2 protein was used to prevent experimentally-induced stenosis. Levels of radiolabelled FGF-2 in the myocardium of pigs treated with retrograde were almost twice the levels achieved using antegrade infusion. Additionally, significant improvements in transmural blood flow and regional myocardial function were reported when FGF-2 was administered via the retrograde method.

In the area of cell therapy, Suzuki et al. [90] reported effective distribution of labeled skeletal muscle progenitor cells via the retrograde method in a rat infarct model. At 28 days increases in cardiac function, decrease in fibrosis, and retention of administered cells was reported. These data suggested the feasibility of the retrograde method for delivery of cells. In a large animal model, George et al. [91], applied the similar technique in a 90-minute occlusion ischemia porcine model. Retrograde venous administration versus antegrade intracoronary administration of bone marrow mononuclear cells resulted in almost double the cell retention. These results were subsequently replicated [92].

To date, only one clinical trial has been reported using the retrograde technique for cell therapy administration. Tuma et al. treated 14 patients with chronic refractory angina using autologous bone marrow mononuclear cells. Cell delivery was successful in all patients with no arrhythmias, elevated cardiac enzymes or complications related to the delivery. All but one patient improved by at least one Canadian Cardiovascular Society class at 2 year follow-up compared to baseline. The median baseline area of ischemic myocardium by SPECT of 38.2% was reduced to 26.5% at one year and 23.5% at two years [93].

Thus in terms of gene [84-88,94], protein [89], and cell delivery [90-93], it appears that retrograde administration via the coronary sinus is superior in terms of safety and efficacy to the intracoronary and possibly intramyocardial routes of administration.

### Applying retrograde administration to ERC therapy: the recover-ERC trial

Medistem has launched the RECOVER-ERC trial in January 2012 to assess the safety and efficacy of ERCs in patients with Congestive Heart Failure (CHF) using a minimally invasive Retrograde Coronary Sinus Delivery. The study is being conducted under the leadership of Dr. Leo Bockeria and Dr. Amit Patel at the Bakoulev Center for Cardiovascular Surgery of Russian Academy of Medical Science. This study is currently enrolling a total of 60 ischemic and non-ischemic congestive heart failure (CHF) patients. Diagnosis of CHF is based on cardiac function tests, cardiac patient assessment, and cardiac physician assessments, which include symptomatic heart failure in NYHA classification, stage III/IV; left ventricular ejection fraction of ≤40% and no other cardiac intervention options likely to improve clinical status. The study includes three escalating dose cohorts. Each consists of 15 treatment (receiving ERCs) patients and 5 controls (receiving carrier-solution, a placebo). After 20 patients are enrolled into a lower dose cohort, the dose escalation in the next cohort for the treatment patients is as follows: 50 million cells (the starting cohort), and then 100 million cells (second cohort), and 200 million cells (third cohort).

Safety endpoints are assessed throughout the study which consist of: Major Cardiac Adverse Events; Adverse Events/Serious Adverse Events; Elevation of Cardiac Enzymes post-infusion; Complete blood count; Physical assessment/vital signs; ECG; and ECHO, MRI or SPECT Abnormalities.

Efficacy endpoints are collected at following time points and compared to baseline values:

•Cardiac function at 3 month, 6 month and 1 year

•Cardiac remodeling as measured by the change in LVEDV compared to baseline measured by echocardiography

•NYHA / CCS Classification at 3 month, 6 month, and 1 year measured by the change in NYHA from baseline; and change in CCS from baseline

•Quality of Life Assessment at 3 month, 6 month, and 1 year measured by the change in Minnesota Living with Heart Failure Questionnaire (MLHFQ) from baseline

Subjects who complete the 6 month study duration will be considered to have completed the study. All subjects should however be followed until completing the study follow-up at 1 year after treatment or until study discontinuation for other reasons.

### Study update

In June 2012, the Data Safety Monitoring Board completed 2 month safety review of the first 5 patients recruited in the study. Based on lack of adverse effects, the study was allowed to continue enrollment. To date 17 patients have been enrolled in the study, with no Serious Adverse Events triggering Data Safety Monitoring Board actions.

## Conclusions

Cardiac cell therapy is currently limited by efficacy and lack of scalable delivery methods. The ERC is a first in class universal donor stem cell, which possesses superior angiogenic properties as compared to other clinical stem cell types. Medistem has initiated a Phase II clinical trial in CHF combining an “off the shelf” cell drug, with a practical delivery system. Success of the trial will position Medistem to spearhead the development of a true cardiovascular regenerative therapy.

## Competing interests

VB, and TEI are shareholders and board members of Medistem Inc. (PINKSHEETS:MEDS). The other authors declare no competing interests.

## Authors’ contribution

LB, ANP, TL, BA, and OB were involved in trial design and execution. LB, VB, OB, TL, BA, EJW, AAB, TEI, and ANP reviewed the literature, wrote the paper, and proofread the final copy. All authors read and approved the final manuscript.
